# Sequential or alternating administration of docetaxel (Taxotere®) combined with FEC in metastatic breast cancer: a randomised phase II trial

**DOI:** 10.1038/sj.bjc.6600165

**Published:** 2002-03-04

**Authors:** M Spielmann, M Tubiana-Hulin, M Namer, H Mansouri, P h Bougnoux, N Tubiana-Mathieu, V Lotz, J C Eymard

**Affiliations:** Institut Gustave Roussy, 39-53 rue Camille Desmoulins, 94805 Villejuif, France; Centre René Huguenin, 35 rue Dailly, 92210 St Cloud, France; Centre A Lacassagne, 33 avenue de Valombrose, 06189 Nice Cedex 02, France; Hôpital Bretonneau, 2 bld Tonnellé, 37044 Tours Cedex 1, France; CHU Dupuytren, 2 avenue Martin Luther King, 87042 Limoges, France; Laboratoire Aventis, 46 Quai de la Rapée, 75601 Paris Cedex 12, France; Centre Jean Godinot, 1 avenue du Général Koenig BP 171, 51056 Reims Cedex, France

**Keywords:** metastatic breast cancer, sequential therapy, alternating therapy, docetaxel, FEC

## Abstract

The aim of this study, using a Fleming single-stage design, was to explore the efficacy and safety of Taxotere® 100 mg m^−2^ docetaxel and FEC 75 cyclophosphamide 500 mg m^−2^, fluorouracil 500 mg m^−2^ and epirubicin 75 mg m^−2^, in alternating and sequential schedules for the first-line treatment of metastatic breast cancer. One hundred and thirty-six women were randomly allocated, to one of three treatment regimens: DTX 100 plus FEC 75, alternated for eight courses (ALT); four courses of DTX 100 followed by four courses of FEC 75 (SEQ T); or four courses of FEC 75 followed by four courses of DTX 100 (SEQ F). One hundred and thirty-one women were evaluable for tumour response. Although the treatment outcome was equivalent in the two sequential arms and the alternating regimen (*P*=0.110, not significant), the response rate was less encouraging in the SEQ F arm (52.3%) than in the other two arms (71.1% for ALT and 70.5% for SEQ T), in which docetaxel was administered first. Time to progression was similar in the ALT, SEQ T and SEQ F arms (9.5, 9.3 and 10.4 months respectively). Grade 3–4 neutropenia was observed in nearly all patients; febrile neutropenia occurred in 9% (ALT), 16% (SEQ T) and 2% (SEQ F) of patients. Few patients (⩽9%) developed grade 3–4 non-haematological toxicities. Relative dose intensity was 97–99% for all regimens. All treatment regimens were active and well tolerated.

*British Journal of Cancer* (2002) **86**, 692–697. DOI: 10.1038/sj/bjc/6600165 www.bjcancer.com

© 2002 Cancer Research UK

## 

Despite recent advances in cancer therapies, metastatic breast cancer (MBC) still carries a poor prognosis. About 40% of patients with breast cancer will develop metastatic disease, either at diagnosis (about 7% of patients) (Wingo *et al*, 1995) or, more commonly, at the time of recurrence. Furthermore, median survival remains at about 2–3.5 years after documentation of metastasis (Clark *et al*, 1987; Henderson, 1991; Campora *et al*, 1999).

The main aim of chemotherapy in MBC is palliative. However, chemotherapy can also improve survival and may enhance quality of life (Fossati *et al*, 1998). The anthracyclines have long been considered to be the most active agents in MBC, with reported response rates of about 50% for monotherapy (Findlay and Walker-Dilks, 1998; Ormrod *et al*, 1999). Of the newer agents now available, docetaxel (Taxotere®), a semi-synthetic taxoid, has shown the most promising activity of all the compounds used so far in MBC, even in anthracycline-resistant tumours (Van Oosterom, 1995; Bonneterre *et al*, 1999; Chan *et al*, 1999; Nabholtz *et al*, 1999; Sjöström *et al*, 1999). Several phase III trials have now established docetaxel as the most active single agent in MBC (Chan *et al*, 1999; Nabholtz *et al*, 1999).

The failure of chemotherapy to produce a cure in MBC is thought to be linked to the development of drug resistance by the tumour cells. One strategy, aimed at overcoming this phenomenon, involves the use of non-cross-resistant chemotherapy regimens. Cyclophosphamide plus methotrexate and fluorouracil (CMF) was one of the first combinations to be investigated in breast cancer. Response rates of between 50 and 60% have been reported (Bonadonna *et al*, 1995). The addition of doxorubicin to combination regimens in MBC is associated with improved response rates (to between 60 and 80%) in advanced breast cancer (A'Hern *et al*, 1993; Fossati *et al*, 1998). However, no survival benefit has been demonstrated (Fossati *et al*, 1998). Commonly used first-line anthracycline-containing regimens involve the addition of either doxorubicin or epirubicin to fluorouracil and cyclophosphamide (FAC or FEC, respectively) (French Epirubicin Study Group, 1988; Italian Multicentre Breast Study with Epirubicin, 1988).

However, improvements in tumour response rates achieved by combination therapies are frequently hampered by an increase in toxicity (Fossati *et al*, 1998). It has been suggested that toxicity can be reduced if different components of chemotherapeutic regimens are administered either alternately (Goldie *et al*, 1982) or sequentially (Day, 1986). To date, few studies have been conducted using alternating or sequential regimens or both in MBC.

The aim of the present study was to evaluate the effect of adding docetaxel to the FEC regimen in the first-line treatment of MBC, and to determine whether the use of either an alternating or a sequential schedule could alter the efficacy or cumulative toxicity of the combination.

## MATERIALS AND METHODS

### Patient selection

Between September 1996 and March 1998, 136 women aged ⩽18 years and <75 years, with histologically or cytologically confirmed MBC, were recruited into the study. Patients with measurable MBC (bidimensionally measurable lesions: nodes or skin and subcutaneous metastases ⩾10×10 mm, lung metastases ⩾10×10 mm on chest X-ray or 20×10 mm on CT scan, and at other sites ⩾20×10 mm on CT scan) were eligible for the study. Prior chemotherapy, either in the neoadjuvant or adjuvant setting, was permitted, provided that relapse had occurred more than 12 months after completion of the chemotherapy. Prior anthracycline-containing chemotherapy was permitted if the cumulative dose of doxorubicin was ⩽300 mg m^−2^ (or ⩽450 mg m^−2^ epirubicin, or ⩽75 mg m^−2^ mitoxantrone). Prior hormone therapy was also permitted, provided it was discontinued at entry into the study. Prior radiotherapy was permitted for palliative purposes, except if the irradiated site was involved in disease evaluation. Radiotherapy was required to be completed at least 4 weeks before study entry and previously irradiated lesions were not evaluable, except in the case of disease progression.

All patients were required to have a WHO performance status of ⩽2; adequate bone marrow function (granulocyte count ⩾2×10^9^ l^−1^, platelet count ⩾100×10^9^ l^−1^); adequate renal function (serum creatinine level ⩽135 μmol l^−1^, or creatinine clearance >60 ml min^−1^); adequate liver function (bilirubin level ⩽1.25 times normal level, ASAT and ALAT levels ⩽2 times normal level, alkaline phosphatases <2.5 times normal level). In addition, all patients were required to have a resting left ventricular ejection fraction of ⩾50% of normal values, or greater than the lower limit of normal (on echocardiographic or radionucleotide scan).

Exclusion criteria included: prior chemotherapy with taxoids (paclitaxel, docetaxel), or contra-indications to corticosteroid therapy; local relapse after conservative surgery alone, or advanced inoperable localised breast cancer; and CNS metastases. Bone metastases, pleural effusion or ascites, or carcinomatous lymphangitis were not permitted as the only evidence of metastatic disease. Patients who had received prior chemotherapy for metastatic disease were not eligible. Additional exclusion criteria were: unstable heart disease requiring treatment; uncontrolled infectious disease; psychiatric or neurological disorders; a history of neoplasm other than breast cancer, with the exception of non-melanoma skin cancer or curatively treated *in-situ* cervical cancer.

The protocol was approved by an ethical committee and all patients provided written, informed consent before study entry.

### Randomisation and study treatments

Registration forms were faxed to Aventis Pharma (Montrouge, France) and the treatment schedule was allocated according to a randomisation list created electronically. Patient groups were stratified by centre and allocated in equal proportion to the three treatments.

Patients who fulfilled the eligibility criteria were randomised to receive either alternating therapy with docetaxel (DTX) and FEC for eight cycles (ALT), or four cycles of DTX followed by four cycles of FEC (SEQ T), or four cycles of FEC followed by four cycles of DTX (SEQ F). Each regimen was repeated every 21 days. DTX 100 mg m^−2^ was administered by 1-h infusion; the premedication schedule consisted of oral prednisolone 50 mg given 13, 7 and 1 h before DTX, 50 mg on the evening of the infusion, and 50 mg twice a day for 3 days after infusion (for a total of 10 doses). The calculated dose of DTX was diluted in 5% dextrose or normal saline to produce a maximal concentration of 1 mg ml^−1^. Vital signs were monitored before and during DTX administration.

The FEC regimen was administered on day 1 of each treatment cycle and consisted of an intravenous (i.v.) infusion of cyclophosphamide 500 mg m^−2^, an i.v. bolus of fluorouracil 500 mg m^−2^, and an i.v. infusion of epirubicin 75 mg m^−2^. Epirubicin was reconstituted with 0.9% normal saline or sterile water for injection to produce a maximal final concentration of 2 mg ml^−1^. Epirubicin was given as a 30–60 min infusion. Fluorouracil and cyclophosphamide were diluted in 5% dextrose or normal saline. Vital signs were monitored before and during FEC administration.

### Toxicity and dose modifications

Toxicity was evaluated and graded according to the National Cancer Institute Common Toxicity Criteria.

The doses of both DTX and epirubicin were adjusted in the event of febrile neutropenia, or when a delay of >7 days was necessary for the absolute neutrophil count to recover to ⩾0.5×10^9^ l^−1^. A platelet count of ⩾100×10^9^ l^−1^ was also necessary for treatment to be recommenced. In these circumstances, DTX was reduced to 75 mg m^−2^, and epirubicin to 50 mg m^−2^. Systematic prophylactic administration of granulocyte colony-stimulating factor (G-CSF) was not permitted. Treatment of neutropenic complications was left to the discretion of the clinician.

In the event of nausea and vomiting of grade ⩾3, and/or mucositis of grade 3/4, the dose of DTX was reduced to 75 mg m^−2^, then to 55 mg m^−2^, as necessary, and the epirubicin dose was reduced to 50 mg m^−2^. If other grade 3 toxicities were reported (excluding alopecia and anaemia) treatment was delayed for a maximum of 2 weeks, until resolution to grade 1 or lower. Treatment was then restarted with the dose reductions described. If treatment was required to be delayed for longer than 2 weeks, the patient was withdrawn from the study.

### Patient and treatment evaluation

A complete medical history and physical examination with tumour measurement were performed in all patients and before each cycle of treatment. Complete blood cell counts with platelet counts were performed weekly, and biochemical parameters were assessed every 3 weeks.

The type of imaging procedures used depended on the tumour location and imaging was repeated after the second, fourth, sixth, and eighth treatment cycles. Each objective response was confirmed at least 4 weeks after initial detection and was reviewed by a panel of independent radiologists.

The duration of partial response was calculated from the start of treatment until the first documentation of progressive disease, while the duration of complete response (CR) was from the first time the CR was documented. Time to first response and time to progression were calculated from the first administration of the drugs to the first occurrence of response and first progression, respectively. Survival was calculated from the date of the first administration of the study drugs to death.

### Study design

The study was designed using Fleming's one-sample multiple testing procedure for phase II clinical trials (Fleming, 1982). This procedure allows for the pre-specification of a minimum response rate to treatment (i.e. one below which the treatment would be considered insufficiently active), as well as the pre-specification of a response rate above which the treatment would be considered sufficiently active. The Fleming study design is thus not intended to provide for a statistical comparison between treatment arms: rather, it is a vehicle that allows investigators to examine efficacy in a number of different treatment arms, and, if necessary, to then curtail one or more of these arms prematurely, if patients do not reach a pre-specified level of response. Thus, the current study was a prospective, open, single-centre, phase II study to assess the efficacy and safety of docetaxel plus FEC, in three administration schedules, in patients with MBC. The primary endpoint was the efficacy of treatment, defined as overall response rate, duration of response, time to progression and overall survival. The secondary objective was to compare the safety profile of the three treatment regimens.

### Statistical analysis and calculation of the sample size

The required number of patients was determined according to a single stage Fleming design (Fleming, 1982). The statistical hypotheses were the same for the three treatment arms. That is to say, the design parameters that were used considered a treatment to be insufficiently active if the response rate was ⩽45%, and sufficiently active if the response rate was ⩾60%. With an error rate of 10% and a power of 73%, 40 evaluable patients needed to be included in each of the three arms. Any statistical analysis was performed on the intention-to-treat group.

## RESULTS

### Patient characteristics

The patient and tumour characteristics were well balanced between the three treatment groups, except that the combined incidence of liver and bone involvement was lower in the SEQ F arm than the other two arms (Table 1

**Table 1:**
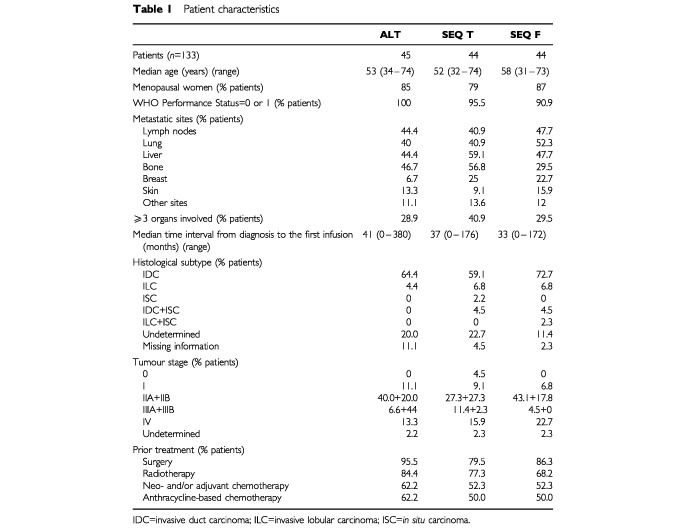
Patient characteristics

). Among the 136 patients who entered the study, 133 were treated and 131 were evaluable for efficacy. The reasons for non-treatment were: withdrawal of consent (*n*=1), suicide (*n*=1), and biological abnormalities (*n*=1). Five patients were not evaluable for efficacy because they received fewer than two cycles of chemotherapy (*n*=4), and/or their tumours were not assessed.

About half of the patients had already received previous anthracycline-based chemotherapy (adjuvant and/or neoadjuvant). Overall median time interval from diagnosis to the first infusion was 39 months (range 0–380 months).

### Treatment exposure

The majority of patients completed the eight courses of planned therapy: 87.6% in the ALT arm, 81.8% in the SEQ T arm, and 75.0% in the SEQ F arm (Table 2

**Table 2:**
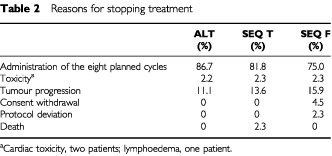
Reasons for stopping treatment

). Two per cent of patients in each arm stopped treatment due to toxicity. Tumour progression was responsible for cessation of therapy in 11.1% in the ALT arm, 13.6% in the SEQ T arm, and 15.9% in the SEQ F arm.

Most cycles in all treatment groups were administered at the initially planned time and dose. Consequently, the relative dose intensity (RDI) was excellent for both drugs: the RDI for docetaxel was 99% (95% CI: 66–105%) in the ALT arm, 97% (95% CI: 75–103%) in the SEQ T arm, and 97% (95% CI: 76–103%) in the SEQ F arm; the RDI for epirubicin was 97% (95% CI: 53–108%) in the ALT arm, 95% (95% CI: 64–102%) in the SEQ T arm, and 96% (95% CI: 71–103%) in the SEQ F arm.

### Efficacy

The overall response rate (complete plus partial responses) was high in the three groups, but higher in the ALT (71.1%) and SEQ T (70.5%) arms than in the SEQ F arm (52.3%) (Table 3

**Table 3:**
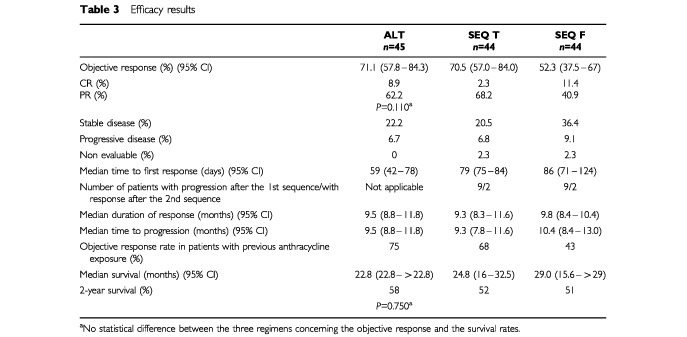
Efficacy results

).

Complete responses were observed in 8.9% of patients in the ALT arm, 2.3% in SEQ T and 11.4% in the SEQ F arm. There were more cases of stable disease in the SEQ F arm. Median duration of response and median time to progression were similar in the three groups (Table 3).

The 2-year survival was similar in the three treatment groups, with a 58% survival rate in the ALT arm, 52% for the SEQ T arm, and 51% for the SEQ F arm. Note that the Fleming study design is not intended to differentiate statistically between the efficacies and the survival rates of these three arms, hence the *P*-values (Table 3) are non-significant.

Median duration of survival was also similar in the three groups. One patient died from progressive disease during the study (SEQ T arm). A further 10 patients died from disease progression after the end of therapy (three patients in the ALT arm, three in the SEQ T arm and four in the SEQ F arm).

The median follow-up period for the whole study population was 19.4 months (range 0.4–34.2 months).

### Safety

There were no deaths due to toxicity during the study. There was no difference in terms of toxicity between the three arms (Tables 4

**Table 4:**
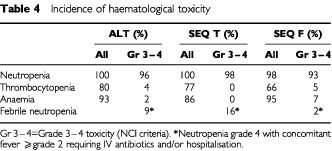
Incidence of haematological toxicity

and 5

**Table 5:**
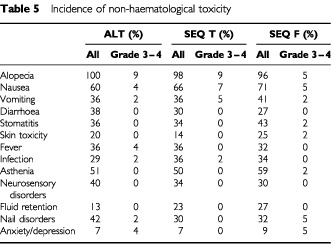
Incidence of non-haematological toxicity

). However, 9% of patients in the ALT arm, 16% in the SEQ T arm and 2% in the SEQ F arm experienced febrile neutropenia. There was only one documented infection with febrile neutropenia during one FEC course in the ALT arm. Four patients received G-CSF for grade IV neutropenia. However, these four patients did not receive prophylactic G-CSF in subsequent cycles.

Non-haematological toxicity, grades 3 and 4, was infrequent in all treatment groups (⩽9% of patients). Grade 3 or 4 fluid retention or neurosensory disorders were not observed in any of the treatment arms.

## DISCUSSION

The present study is the first to evaluate the efficacy and tolerability of one of the recently available taxoids in alternating and sequential schedules for the treatment of MBC.

Several phase III trials have established docetaxel as the most active single agent in MBC. A recent large randomised trial demonstrated improved survival with docetaxel compared with combination therapy with mitomycin C plus vinblastine in patients with MBC who had progressed despite prior anthracycline-containing chemotherapy (Nabholtz *et al*, 1999). In another multicentre, randomised phase III trial a higher response rate was observed with docetaxel (47.8%) compared with doxorubicin (33.3%) in patients with MBC who had received prior alkylating agent-containing chemotherapy (Chan *et al*, 1999).

In an effort to overcome the possible development of drug resistance by the tumour cells, non-cross-resistant combination regimens have been used. Doxorubicin or epirubicin have been added to fluorouracil and cyclophosphamide (FAC or FEC, respectively). These regimens appear to have equivalent efficacy in MBC, but several studies have shown that the FEC regimen is more convenient to administer than FAC, and is also associated with lower toxicity than FAC (French Epirubicin Study Group, 1988; Italian Multicentre Breast Study with Epirubicin, 1988). These studies reported response rates with the FEC regimen of 43 to 64%. Furthermore, increasing the dose of epirubicin from 50 to 75 mg m^−2^ appears to improve the response rates in patients with advanced breast cancer who are treated with the FEC regimen, with no significant effect on tolerability (French Epirubicin Study Group, 1991; Ormrod *et al*, 1999).

Docetaxel has been associated with the development of myelotoxicity (Fumoleau, 1997) and epirubicin is also known to be haematotoxic (Ormrod *et al*, 1999). Therefore, the combination of these agents may be expected to produce additive haematotoxic effects. Furthermore, epirubicin produces a cumulative cardiac toxicity, especially at cumulative doses above 900 mg m^−2^ (Ryberg *et al*, 1998; Gennari *et al*, 1999). It has been suggested that toxicity can be reduced if different components of combination regimens are administered either alternately (Goldie *et al*, 1982) or sequentially (Day, 1986). A number of studies have investigated the role of alternating or sequential regimens in the treatment of MBC. Most of these trials appear to indicate no clear advantage of sequential over alternating regimens.

A randomised study comparing sequential to alternating doxorubicin and CMF therapy in the adjuvant treatment of patients with breast cancer and more than three positive axillary nodes has already been published (Bonadonna *et al*, 1995). The sequential regimen was shown to be significantly superior to the alternating one, in terms of both relapse rates (42% *vs* 28%, *P*=0.002) and overall survival (58% *vs* 44%, *P*=0.002). These results influenced the design of subsequent clinical studies and may explain the large number of ongoing studies that are evaluating sequential regimens in the adjuvant setting (Hudis *et al*, 1999; Burris, 2000; Di Leo *et al*, 2000). It would appear, therefore, that further evaluation of sequential and alternating regimens is necessary in MBC.

In the study reported here, which uses a Fleming one-stage design (Fleming, 1982), with pre-specified parameters for criteria the investigators would consider to be acceptable response rates, it is interesting to note that no statistical difference in the treatment outcome was demonstrated between the two sequential arms and the alternating regimen (*P*=0.110, not significant). However, the response rate was less encouraging in the SEQ F arm (52.3%) than in the other two arms (71.1% for ALT and 70.5% for SEQ T), in which docetaxel was administered first, despite a lower combined incidence of bone and liver metastases among patients in the SEQ F arm. A poor response rate to chemotherapy is generally observed in bone metastases and the efficacy of treatment is often difficult to determine at this site (Leonard *et al*, 1994). Furthermore, metastases in the liver are known to be less chemosensitive than those at other sites.

None of the tested schedules was clearly superior in terms of toxicity. The majority of cycles of the three regimens was administered at the planned dose-intensity and was well tolerated, with no cumulative toxicities being observed.

In conclusion, given that the aim of the present study was to evaluate the effect of adding docetaxel to the FEC regimen for the treatment of MBC, and to determine whether the use of either an alternating or a sequential schedule could alter the efficacy or cumulative toxicity of this combination, our results indicate that the addition of docetaxel to the FEC regimen is, indeed, active in MBC, and that the ALT and SEQ T regimens warrant further investigation.
